# Diet and exercise signals regulate SIRT3 and activate AMPK and PGC-1α in skeletal muscle

**DOI:** 10.18632/aging.100075

**Published:** 2009-08-15

**Authors:** Orsolya M. Palacios, Juan J. Carmona, Shaday Michan, Ke Yun Chen, Yasuko Manabe, Jack Lee Ward III, Laurie J. Goodyear, Qiang Tong

**Affiliations:** ^1^ USDA/ARS Children's Nutrition Research Center, Department of Pediatrics, Baylor College of Medicine, Houston, TX 77030, USA; ^2^ Howard Hughes Medical Institute & Paul F. Glenn Laboratories for the Biological Mechanisms of Aging, Department of Pathology, Harvard Medical School, Boston, MA 02115, USA; ^3^ Massachusetts General Hospital Cancer Center, Charlestown, MA 02129, USA; ^4^Department of Society, Human Development, and Health, Harvard School of Public Health, Boston, MA 02115, USA; ^5^ Paul F. Glenn Laboratories for the Biological Mechanisms of Aging, Department of Pathology, Harvard Medical School, Boston, MA 02115, USA; ^6^ Joslin Diabetes Center & Brigham and Women's Hospital, Harvard Medical School, Boston, MA 02115, USA; ^7^ These authors contributed equally to this work

**Keywords:** SIRT3, AMPK, PGC-1α, caloric restriction, skeletal muscle, exercise, high-fat diet

## Abstract

SIRT3 is a member of the sirtuin family of NAD^+^-dependent deacetylases, which is localized to the mitochondria and is enriched in kidney, brown adipose tissue, heart, and other metabolically active tissues. We report here that SIRT3 responds dynamically to both exercise and nutritional signals in skeletal muscle to coordinate downstream molecular responses. We show that exercise training increases SIRT3 expression as well as associated CREB phosphorylation and PGC-1α up-regulation. Furthermore, we show that SIRT3 is more highly expressed in slow oxidative type I soleus muscle compared to fast type II extensor digitorum longus or gastrocnemius muscles. Additionally, we find that SIRT3 protein levels in skeletal muscle are sensitive to diet, for SIRT3 expression increases by fasting and caloric restriction, yet it is decreased by high-fat diet. Interestingly, the caloric restriction regimen also leads to phospho-activation of AMPK in muscle. Conversely in SIRT3 knockout mice, we find that the phosphorylation of both AMPK and CREB and the expression of PGC-1α are down regulated, suggesting that these key cellular factors may be important components of SIRT3-mediated biological signals in vivo.

## Introduction

The sirtuin family of proteins possesses NAD^+^-dependent deacetylase activity and/or ADP ribosyltransferase activity. The seven mammalian sirtuins (SIRT1-7) sirtuins (SIRT1-7) are localized differentially within the cell and have a variety of functions [1,2]. SIRT1 is the most extensively studied member of the family and regulates diverse biological processes ranging from DNA repair and genome stability to glucose and lipid homeostasis [3,4]. Although three specific sirtuins, SIRT3-5, are found in the mitochondria [5,6], not much is known about their function *in vivo *[7]. SIRT4 has been shown to regulate amino acid-stimulated insulin secretion by targeting glutamate dehydrogenase [8], and it was recently demonstrated that SIRT5 participates in the urea cycle [9]. Among the mitochondrial sirtuins, SIRT3 possesses the most robust deacetylase activity [10-12]. Indeed, significantly higher levels of mitochondrial protein acetylation were detected in the livers of SIRT3-null mice, compared to those of SIRT4 or SIRT5 knockout animals [13]. However, little is known about the physiological role of SIRT3 despite the fact that a number of SIRT3 substrates and co-precipitating proteins have been identified: acetyl-CoA synthetase 2 [14], Ku70 [15], FOXO3a [16], subunit 9 of mitochondrial Complex I(NDUFA9) [17], glutamate dehydrogenase [13,18] and isocitrate dehydrogenase 2 [18].

SIRT3 has been linked to longevity in men [19,20] and aberrant expression of this sirtuin correlates with node-positive breast cancer in clinical biopsies from women [21]—suggesting that SIRT3 serves as an important diagnostic and therapeutic target in human health/aging and disease, affecting men and women in unique ways. In human cells, we have shown that SIRT3, along with SIRT4, is required for Nampt-mediated cell survival after genotoxic stress, wherein maintenance of mitochondrial NAD^+^ levels inhibits apoptosis [22]. Previously we also reported in murine brown adipose tissue that the RNA level of SIRT3 increases by cold exposure and caloric restriction (CR) and that constitutive expression of SIRT3, in brown pre-adipocytes, stimulates downstream CREB-mediated expression of PGC-1α and other mitochondrial-related genes [10]. In this study, we investigated the physiological conditions that regulate SIRT3 in skeletal muscle, a metabolically active organ vital for insulin-mediated glucose disposal and lipid catabolism. Notably, skeletal muscle strongly influences whole-body lipid metabolism, as lipid catabolism provides up to 70% of the energy usage for resting muscle [23]. In this tissue, the balance between fatty acid availability and fatty acid oxidation rates plays an important role in regulating insulin responses, and intramuscular fatty acid metabolites like diacylglycerol may cause insulin resistance [24]. Therefore, studying the role of molecular factors and pathways acting in muscle under various dietary and environmental conditions will be critical for better understanding metabolism, health, and disease.

In skeletal muscle, the peroxisome proliferator-activated receptor gamma coactivator-1α (PGC-1α), a nuclear receptor co-activator, plays multiple roles in metabolic regulation [25,26]. It stimulates mitochondrial biogenesis [27], induces muscle fiber-type switch, and increases oxidative capacity in skeletal muscle cells [28]. In addition to transcriptional activation by CREB [29], it has been shown that AMP-activated protein kinase (AMPK) also increases PGC-1α expression [30,31] and activates it by direct phosphorylation [32]. AMPK is also a key molecular sensor and regulator of muscle metabolism.

AMPK is a ubiquitous heterotrimeric serine/threonine protein kinase, which functions as a fuel sensor in many tissues, including skeletal muscle [33]. AMPK is allosterically activated by AMP and by phosphorylation at Thr172 in the catalytic α-subunit, mainly by an upstream AMPK kinase, LKB1 [34,35]. Importantly, AMPK is stimulated by cellular stresses that deplete ATP and elevate AMP, such as diet restriction/hypoglycemia [36], exercise [37], and muscular contraction [38]. Activated AMPK stimulates ATP-generating catabolic pathways, such as cellular glucose uptake and fatty acid α-oxidation. AMPK activation also represses ATP-consuming processes, such as lipogenesis, to restore intracellular energy balance [33,39].

Our work seeks to further elucidate the role of sirtuins within health and disease, with particular focus on muscle tissue in this study. We report here that expression of SIRT3 in skeletal muscle is sensitive to various signals from both diet and exercise, leading to downstream activation of AMPK and up-regulation of PGC-1α. SIRT3 is therefore a potential key regulator of skeletal muscle biology, responding to important environmental cues and activating cellular factors *in vivo*.

## Results

### SIRT3 is regulated in skeletal muscle by exercise training

**Figure 1.**
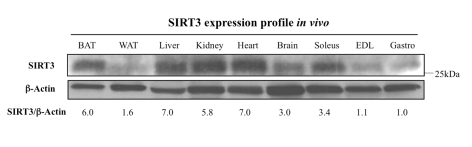
Tissue distribution of SIRT3 protein. The SIRT3 protein is abundantly expressed in the brown adipose tissue (BAT), liver, kidney, heart, brain, and soleus muscle, but very low in white adipose tissue (WAT), the extensor digitorum longus muscle (EDL), or the gastrocnemius muscle (Gastro). For each sample, 50 μg of protein was loaded into a 10% acrylamide gel, electrophoresed, and transferred to a nitrocellulose membrane. The membrane was probed using an anti-SIRT3 serum or an anti-β-actin antibody. Blots were quantified with ImageQuant and SIRT3/actin ratios are provided; since gastrocnemius (Gastro) has the lowest SIRT3 expression *in vivo*, normalization (l.0) was set with respect to this tissue.

We first assayed the SIRT3 expression profile *in vivo* to compare the whole-body distribution of SIRT3, specifically across muscles to tissues like adipose and kidney, where SIRT3 has been previously described. As predicted, the SIRT3 tissue distribution pattern mirrors that of SIRT3 mRNA [11]. Indeed, SIRT3 exhibits high expression in important metabolically active tissues like kidney, brown fat, liver, and brain (Figure 1). When comparing expression across muscle samples, we noticed that SIRT3 protein levels were higher in the slow-twitch soleus muscle compared to the fast-twitch muscles like extensor digitorum longus and gastrocnemius, in agreement with higher mitochondrial content and the oxidative feature of the soleus muscle.

**Figure 2.**
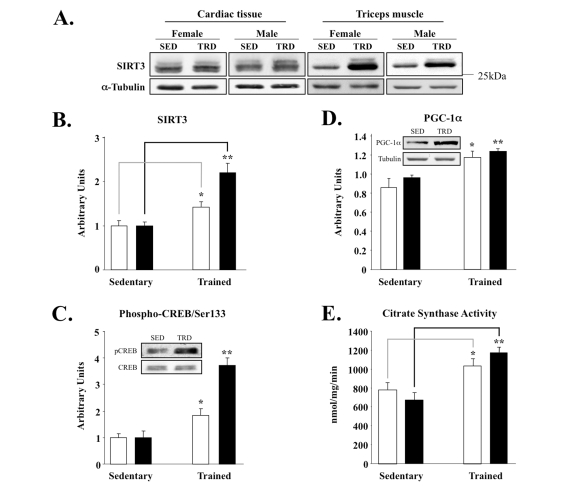
Skeletal muscle-specific induction of SIRT3 and associated factors in exercise-trained mice. (**A**) Triceps or cardiac muscle tissue was homogenized and 50 μg of protein was analyzed by Western blot, using anti-SIRT3 serum (Covance) and α-tubulin control; representative blots are shown here and throughout. SED = sedentary and TRD = trained. (**B**) Quantification of SIRT3 band intensities using ImageQuant from blots with animals grouped by sex. Males are plotted as clear bars and females as shaded bars. Total number of animals used per cohort and graphed are as follows: sedentary males, N = 7; sedentary females, N = 5; exercised males, N = 8; exercised females, N = 6. (**C**) Phospho-CREB/Ser133 and total CREB protein. Band intensities of phospho-CREB and CREB were quantified and phospho-CREB content was normalized relative to total CREB content; inset provides sample blots of male triceps tissue. (**D**) Induction of PGC-1α correlates with enhanced SIRT3 expression in triceps; samples processed and analyzed, as above. Inset blots are of male triceps tissue. (**E**) Citrate synthase activity was measured as a mitochondrial marker from the same triceps samples, as described previously [40]. N = 2, *P < 0.05, **P < 0.01.

To study the role of SIRT3 in muscle within the context of exercise biology, we next tested if SIRT3 protein levels were sensitive to an established voluntary exercise protocol [40]. Using a specific anti-mouse SIRT3 polyclonal antibody, we found that SIRT3 protein increased selectively in triceps, the muscle that undergoes training in the wheel-caged system, but not in cardiac muscle samples from those same animals (Figure 2A). In contrast to SIRT3, exercise training failed to alter SIRT1 protein levels in triceps (data not shown). The specificity of our antibody for detecting the endogenous ~28kDa SIRT3 protein was confirmed by using SIRT3 knockout tissue lysates (Supplemental Figure 1). Notably, induction of SIRT3 in skeletal muscle was higher in female mice when compared to that of male littermates (Figure 2B). In agreement with this up-regulation, we also observed increased SIRT3 levels in the gastrocnemius muscle of rats exercised on a treadmill-based exercise paradigm [41] (Supplemental Figure 2). Even one week of treadmill training was sufficient to increase SIRT3 protein amount (Supplemental Figure 2B). The up-regulation of SIRT3 (Figure 2B) correlated with enhanced downstream phosphorylation of CREB at Ser133 (Figure 2C) and PGC-1α induction (Figure 2D). Lastly, citrate synthase activity, a mitochondrial marker for exercise training, was significantly higher in trained muscles than in the respective sedentary control group (Figure 2E). Collectively, these data suggest that the up-regulation of SIRT3 by exercise is an important and conserved molecular consequence of training.

### SIRT3 expression in skeletal muscle is sensitive to dietary intake

Previously we had demonstrated how CR stimulates the *in vivo* expression of SIRT3 in brown fat [10]. Thus we hypothesized that perhaps SIRT3 expression in muscles is also sensitive to nutritional signals, especially given how different muscles contain various levels of SIRT3 (Figure 1) and vary inherently with respect to energetic/metabolic potential. To test this hypothesis, we measured the SIRT3 levels in leg muscles of mice in either CR or *ad libitum* (AL) cohorts after twelve months. CR is an effective environmental method known to extend lifespan in a number of model organisms, from yeast and nematodes to rodents, yet the underlying molecular mechanisms by which this pathway acts *in vivo* remain largely unknown [42].

**Figure 3.**
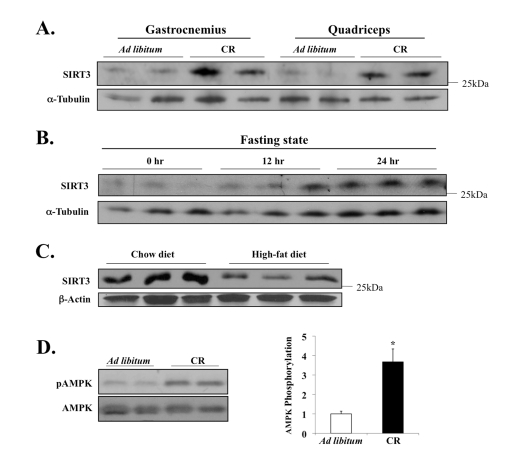
Diet-sensitive expression of SIRT3 and AMPK in muscle tissue. (**A**) Mice were fed NIH-31 standard feed *ad libitum* or NIH-31/NIA-fortified diet (Harlan Teklad) with a daily food allotment of 60% of the control mice to establish caloric restriction (CR); twelve months after the onset of CR, tissues were harvested to examine SIRT3 expression. (**B**) Mice were deprived of food for 24 hours, and SIRT3 level in EDL muscle was determined by Western blot analysis. (**C**) SIRT3 protein expression is decreased in murine hind-leg muscle after 3 months of high-fat diet feeding; total hind-leg tissue protein was isolated and analyzed. (**D**) AMPK T-172 phosphorylation and AMPK total protein in the quadriceps of the caloric restricted mice were assayed; AMPK phosphorylation was determined as phospho-AMPK normalized by total AMPK. N=3, *P < 0.05.

Here we found that the CR diet significantly increased levels of SIRT3 protein in skeletal muscle, compared to the AL control diet (Figure 3A). In addition, twenty four hours of fasting was sufficient to induce the muscle expression of SIRT3 (Figure 3B). Conversely, SIRT3 protein level was significantly decreased following three months of energy-dense, high-fat feeding (Figure 3C), indicating that the SIRT3 expression in muscle fluctuates in response to dietary nutrient uptake. We next measured the effect of CR on AMPK—an enzyme whose activity is dependent on changes in metabolic/energetic potential [30,31].

Since AMPK is activated during decreased energy levels, we hypothesized that AMPK may be activated under CR. In nematodes, for example, it has recently been shown that AMPK is critical for mediating key downstream biological effects that enable lifespan extension by caloric/dietary restriction [43]. Our data here show that AMPK is hyper-phospho-activated at Thr172 of its catalytic α-subunit, which was quantified and determined to be three to four times higher than the AL control diet (Figure 3D). Together these data provided novel connections between caloric intake, SIRT3 and AMPK that merit more analysis.

### Loss of SIRT3 significantly impacts activation of AMPK, CREB and PGC-1α expression

**Figure 4.**
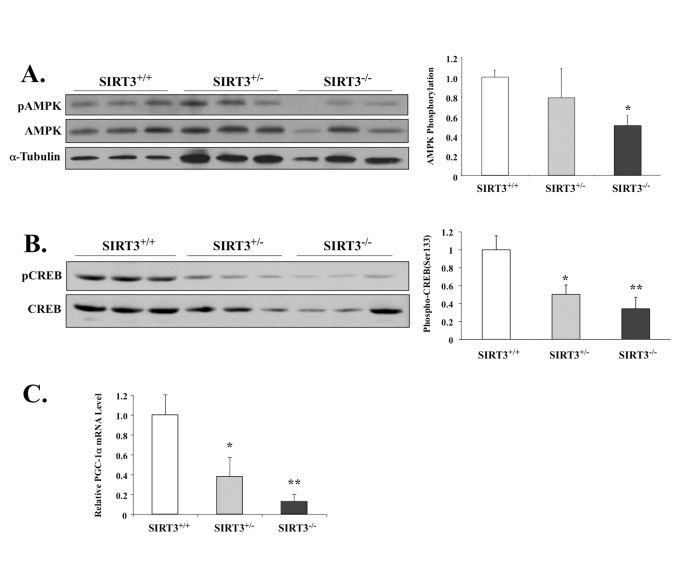
SIRT3-deficient mice have lower phosphorylation levels of AMPK and CREB, as well as decreased PGC-1α mRNA. (**A**) AMPK T-172 phosphorylation and AMPK total protein in the EDL muscles of the male wild-type mice or mice with heterozygous or homozygous SIRT3 gene deficiency were determined by Western blot analysis. AMPK phosphorylation was determined as phospho-AMPK normalized by total AMPK. N=3, *P < 0.05. (**B**) CREB phosphorylation and CREB total protein in the EDL muscles of the wild-type mice or mice with heterozygous or homozygous SIRT3 gene deficiency were determined by Western blotting analysis. CREB phosphorylation was determined as phospho-CREB normalized by total CREB. N=3, *P < 0.05, **P<0.01. (**C**) Quatitative RT-PCR shows pgc-1α mRNA level reduced in the gastrocnemius of SIRT3 knockout mice. *P < 0.05, **P<0.01.

We next tested if the lack of SIRT3 would impact AMPK and other related factors like CREB and/or PGC-1α in skeletal muscle. Consistent with our previous data, we found that SIRT3-null animals had 50% lower levels of AMPK phosphorylation compared to the wild-type littermate control group (Figure 4A). In our exercise model (Figure 2A-D), SIRT3 up-regulation enhanced downstream activation of CREB and PGC-1α. Accordingly, in the SIRT3-null mice, activating phosphorylation of CREB at Ser122 was also reduced (Figure 4B), which correlated with lowered transcriptional activation of *pgc-1*α (Figure 4C). This result is consistent with previously published data, which show that both AMPK and CREB activate *pgc-1*α expression *in vivo* [29].

## Discussion

**Figure 5.**
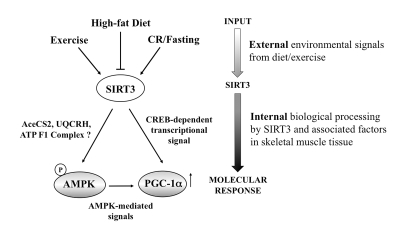
Schematic diagram of potential SIRT3 action in the skeletal myocyte. Collectively, our data support a working model in which SIRT3 responds dynamically to various nutritional and physiological signals to potentially impact muscle energy homeostasis *via* AMPK and PGC-1α**.** Since AMPK can also phosphorylate and activate CREB [52], SIRT3 may activate CREB directly or through AMPK. Given its dynamic role, SIRT3 action within the skeletal muscle cells may serve as an important diagnostic and therapeutic target for impacting human health and disease.

Here we have found that SIRT3 is differentially expressed *in vivo*, with the greatest expression observed in metabolically active tissues like skeletal muscle, where SIRT3 undergoes dynamic regulation by different environmental regimens. SIRT3 protein level is decreased by high-fat feeding, while it is increased by short-term fasting (24-hour) or long-term nutrient deprivation (12-month CR) and exercise training. In this study we also show that loss of SIRT3 significantly inhibits AMPK and CREB phosphorylation, which decreases PGC-1α transcriptional expression in muscle. Consequently, we propose a new model in which SIRT3 leads to potential downstream changes in response to important environmental signals (Figure 5).  This model suggests that SIRT3 levels may respond to various nutritional/energetic and physiological challenges by regulating muscle energy homeostasis *via* factors like AMPK and PGC-1α.

Given our study, it will be interesting to test whether SIRT3-null animals show any defects under certain environmental challenges. Despite the hyper-acetylation of mitochondrial proteins in SIRT3 knockout mice, the significance of these biochemical changes is unclear. A recent study of SIRT3-deficient mice by another group did not find defects in basal metabolism nor adaptive thermogenesis, while the mice were housed in standard dietary/sedentary conditions [13]. Similarly, we found normal treadmill performance in SIRT3 knockout mice while under standard housing (unpublished observations). Upon challenge with various environmental signals, however, these animals may respond differently. Accordingly, we are actively testing how challenges by CR/fasting/high-fat diet and exercise may affect the SIRT3-null mice and alter key downstream cellular factors in muscle cells.

The mechanism(s) by which different environmental variables modulate SIRT3 and activate AMPK in muscle (and other tissues that highly express SIRT3) remains to be fully elucidated. For example, activation of acetyl-CoA synthetase 2 (AceCS2) by SIRT3 [44,45] may elevate the AMP/ATP ratio and consequently activate AMPK. Alternatively, a recent proteomics-based approach has identified many novel SIRT3-interacting partners in human cells, including the ATP synthase (mitochondrial F1 complex) alpha/beta subunits and the ubiquinol-cytochrome c reductase hinge protein, UQCRH [46]. Since these proteins (together with NDUFA9 [17]) serve as critical components of the ATP-generating machinery in cells, SIRT3 may also potentially modulate the AMP/ATP ratio *via* these factors to activate AMPK. Moreover, we too have purified additional putative SIRT3-binding proteins from HeLa cells (using a related cross-linking/immunoaffinity purification method [47]), which include mitochondrial acetoacetyl CoA thiolase (also referred to as α-ketothiolase), malate dehydrogenase, thioredoxin 2 (Trx-2), Hsp60, and lactate dehydrogenase (unpublished data). Since some of these enzymes are important regulators of muscle energy homeostasis, our data further substantiate that SIRT3 may modulate ATP/energy levels *via* key targets to activate AMPK. It is intriguing to note that a study of an independent line of SIRT3-knockout mice indicated that the ATP level is significantly reduced in several tissues [17], although the effect of SIRT3 deficiency on muscle ATP level has not been reported.

Interestingly, it has also been shown that activation of AMPK, upon glucose nutrient restriction of muscle stem cells, causes an increase in the cellular NAD^+^/NADH ratio, consistent with a positive feedback loop needed for prolonged SIRT1 activation [48], as may occur in our SIRT3 model and merits testing. Indeed a second *in vitro* study independently validates a similar NAD^+^/NADH model *via* AMPK [49]. Strikingly, AMPK activation (as occurs with CR) may also result in lifespan extension [50-52], and future study will reveal if SIRT3 is involved in this process. It is known that activated AMPK directly phosphorylates PGC-1α[32] and CREB [53]—and that both AMPK and CREB are involved in the transactivation of PGC-1a [54,55]. Lastly, both SIRT3 and SIRT1 promote mitochondrial biogenesis and fatty acid oxidation *via* PGC-1α but in different ways. SIRT3 promotes PGC-1α expression while SIRT1 activates PGC-1α by direct deacetylation [56]. However, we have found that exercise training regulates SIRT3 but not SIRT1 expression in muscle. At present, it remains to be considered how these two key sirtuin enzymes may work cooperatively within certain tissues in response to environmental signals.

Furthermore, it is important to consider that a phenotype may be tissue-specific, especially if SIRT3 has different biological roles in the body. For example, in the rennin-angiotensin system, which plays a key role in the pathophysiology of cardiac and renal disease in humans, targeted disruption of the angiotensin receptor (*Agtr1a* gene in mice) yields animals with less cardiac and vascular injury, prolonged lifespan, increased number of mitochondria, and dramatic up-regulation of SIRT3 in kidney tissue—a possible site of SIRT3 action that may contribute, at least in part, to the phenotype that is observed [57]. Another interesting place of molecular action is in brown adipose tissue (BAT), in which SIRT3 has been previously shown to respond dynamically to CR and regulate fat cell physiology *via* PGC-1α [10]. With the recent discovery of BAT in humans (reviewed in [58]), there are now new opportunities to explore the role of SIRT3 in diabetes and obesity research [59]. Moreover, after intense swimming, it has been reported that the expression of SIRT3 and PGC-1α increases in white blood cells to activate the antioxidant response [60]. Lastly, in human skeletal muscle, it has been reported that SIRT3 and PGC-1α expression decline with age and correlate with a sedentary proteomic profile found in people with decreased metabolic output [61]. With exercise, however, these authors observed that the effect is reversed. Collectively, these data suggest that SIRT3 function is perhaps varied throughout the body and specialized to meet the unique metabolic/energetic capacities found within various tissues, particularly in response to environmental cues.

Thus it will be interesting to test whether inducible tissue-specific SIRT3-null mice show global metabolic defects from exercise and/or diet regimens in various parts of the body, especially with aging. This inducible genetic approach will also allow us to bypass potential compensatory effects resulting from the lack of SIRT3 during development. Additionally, a mouse model with increased SIRT3 over-expression in muscle (and/or other specific tissues) will also be a valuable tool for further elucidating the biological role(s) of this sirtuin *in vivo*. All of this work will be important as we fight against aging and associated disorders ranging from type 2 diabetes (and other metabolic diseases) to breast cancer, in which expression of SIRT3 is aberrant. Therefore, small-molecule activators of SIRT3, currently in development and testing [62], may provide novel and key therapeutic routes for the treatment of a variety of common diseases, perhaps by mimicking the beneficial molecular effects of exercise and/or caloric restriction *in vivo*.

## Experimental procedures

Animals, diet and exercise. Ethics statement: Protocols for animal use were in accordance with the guidelines of the Institutional Animal Care and Use Committees of Baylor College of Medicine and the Joslin Diabetes Center and the National Institutes of Health. For the caloric restriction experiment, C57BL/6 male mice were singly caged. At 8 weeks of age, control mice were fed *ad libitum* with NIH-31 standard diet (Harlan Teklad), while food consumption was measured daily. Caloric restricted mice were fed with NIH-31/NIA-fortified diet (Harlan Teklad) with a daily food allotment of 90%, 70% and then 60% of the amount consumed by the control mice—at the first, second, and third week, respectively. From then on, daily food allotment stabilized at 60% of *ad libitum* food intake for the caloric restricted mice. 12 months later, mice were dissected to collect tissues for analysis. For the fasting experiment, food was removed from 3 months old C57BL/6 male mice at 6pm for 24 hours. For the high-fat diet feeding experiment, 8-week-old male mice were fed a control diet or a 35% fat-enriched chow (BioServ) for three months. Various tissueswere also harvested from mice fed the control diet to examine SIRT3 gene expression by Western blot analysis at the termination of the study. For the exercise study [40], 7-week-old male and female FVB/NJ mice were wheel-cage trained for 6 weeks and fed PicoLab Mouse Diet 20 (LabDiet/Purina). In brief, mice were housed in individual cages with or without rodent running wheels (Nalgene, Rochester, NY) and the animals could exercise voluntarily during a 6-week training period. At the end of the 6 weeks, mice were euthanized, triceps muscles were removed and subsequently analyzed for SIRT3, CREB, phospho-CREB/Ser122, and PGC-1α protein expression [10]. Citrate synthase activity was measured as a mitochondrial marker post-exercise training from triceps samples, as described previously [40].

*Sirt3 *-knockout mice. Mice in which the *Sirt3 *gene (Accession: NM_022433) was targeted by gene trapping were obtained from the Texas Institute for Genomic Medicine (Houston, TX, USA). Briefly, these mice were created by generating embryonic stem (ES) cells (Omnibank No. OST341297) with a retroviral promoter trap that functionally inactivates one alleleof the *Sirt3* gene, as described previously [63]. Sequenceanalysis indicated that retroviral insertion occurred in the intron preceding coding exon 2 (Supplemental Figure 1). Targeted 129/SvEvBrd embryonic stem cells were injected into C57BL/6 albino blastocysts. The chimeras (129/SvEvBrd) were then crossed with C57BL/6 albinos to produce the heterozygotes. Heterozygotes were then mated and the offspring were genotyped using PCR, containing two primers flanking the trapping cassette insertion site TG0003-5' (ATCTCGCAGATAGGCTATCAGC) and TG0003-3' (AACCACGTAACCTTACCCAAGG), as well as a third primer LTR rev, a reverse primer located at the 5' end of the trapping cassette (ATAAACCCTCTTGCAG TTGCATC). Primer pair TG0003-5' and TG0003-3' amplify a 336bp fragment from the wild-type allele, while primer pair TG0003-5' and LTR rev amplify a 160bp fragment from the knockout allele.

Antibodies and Western blots. The antibodies used for Western blot analysis included: anti-mouse SIRT3 serum raised against the C-terminus (DLMQRERGKLD GQDR, Genemed Synthesis, Inc.) and used for the tissue distribution and high-fat diet analyses; anti-mouse and anti-rat SIRT3 serum was also developed against the C-terminal regions of each respective protein (Covance), and the anti-mouse serum was validated for specificity using brown fat, cardiac tissue, and soleus muscle from SIRT3 knockout mice (Supplemental Fig. 1), then used for analyzing the exercise samples. Other antibodies used included the following: anti-phospho-CREB/Ser133 (Cell Signaling); anti-CREB (Cell Signaling); anti-phospho-AMPK (Cell Signaling); AMPK (Cell Signaling); anti-PGC-1α (Calbiochem); β-actin antibody (Santa Cruz); and α-tubulin (Abcam).

## Supplementary figures

Supplementary Figure 1 Murine Sirt3 gene structure and inactivation. (**A**) Annotated Sirt3 gene structure [62, 52], showing retroviral insertion site for inactivation of SIRT3 in the null mice. Lines indicate relative position of known ATG start codons; the stop codon, TAA, is indicated in exon 7 (E7). Nomenclature for the exon designations shown here is taken from Cooper et al. [52]. (**B**) SIRT3 protein levels were assayed from mice tissues with either homozygous or heterozygous Sirt3 gene deficiency, using standard Western blot analysis (as before).

Supplementary Figure 2 SIRT3 up-regulation by exercise is conserved in rodents. (**A**) Representative Western blot panels of mice muscle samples used for quantification in Figure 2, and (**B**) rat muscle showing that SIRT3 up-regulation occurs as early as 1-week on a previously established treadmill-based exercise paradigm [52]. Remarkably, the molecular size of the mouse and rat SIRT3 proteins is conserved.
