# Supplementary figures and images for: Diet and exercise signals regulate SIRT3 and activate AMPK and PGC-1α in skeletal muscle
Source: Aging (Albany NY). 2009 Aug 15;1(9):771–83. doi: 10.18632/aging.100075 (PMC2815736; doi:10.18632/aging.100075)

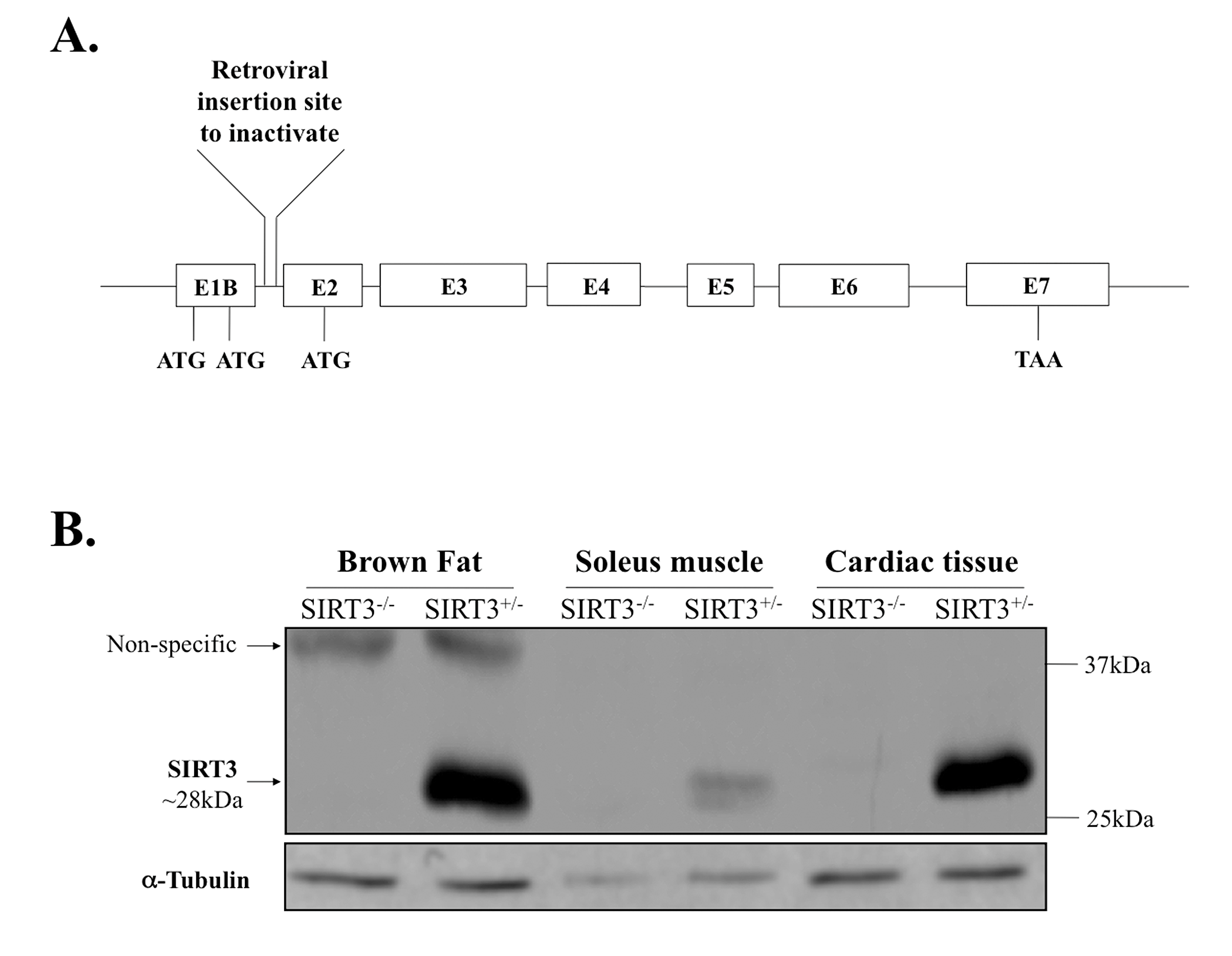

Supplement: Supplementary Figure 1 — (A) Annotated Sirt3 gene structure [62, 52], showing retroviral insertion site for inactivation of SIRT3 in the null mice. Lines indicate relative position of known ATG start codons; the stop codon, TAA, is indicated in exon 7 (E7). Nomenclature for the exon designations shown here is taken from Cooper et al. [52]. (B) SIRT3 protein levels were assayed from mice tissues with either homozygous or heterozygous Sirt3 gene deficiency, using standard Western blot analysis (as before). [file aging-01-771-s001.tif]

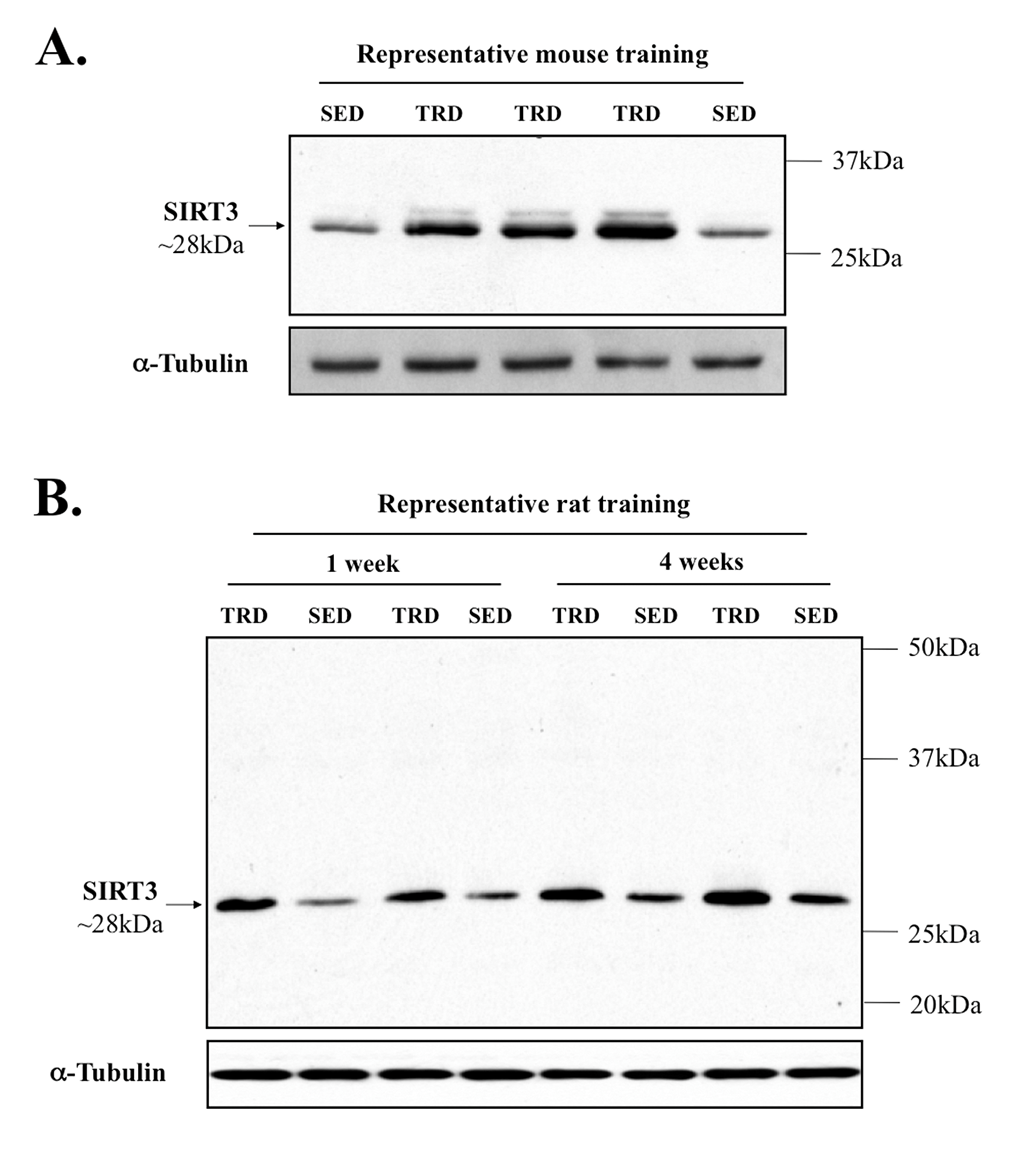

Supplement: Supplementary Figure 2 — (A) Representative Western blot panels of mice muscle samples used for quantification in Figure 2, and (B) rat muscle showing that SIRT3 up-regulation occurs as early as 1-week on a previously established treadmill-based exercise paradigm [52]. Remarkably, the molecular size of the mouse and rat SIRT3 proteins is conserved. [file aging-01-771-s002.tif]
